# Crosstalk between neutrophil extracellular traps and immune regulation: insights into pathobiology and therapeutic implications of transfusion-related acute lung injury

**DOI:** 10.3389/fimmu.2023.1324021

**Published:** 2023-12-07

**Authors:** Yi Liu, Rong Wang, Congkuan Song, Song Ding, Yifan Zuo, Ke Yi, Ning Li, Bo Wang, Qing Geng

**Affiliations:** ^1^ Department of Thoracic Surgery, Renmin Hospital of Wuhan University, Wuhan, China; ^2^ Institute of Hematology, Union Hospital, Tongji Medical College, Huazhong University of Science and Technology, Wuhan, China

**Keywords:** neutrophil extracellular traps, immune regulation, transfusion-related acute lung injury, neutrophil, therapy

## Abstract

Transfusion-related acute lung injury (TRALI) is the leading cause of transfusion-associated death, occurring during or within 6 hours after transfusion. Reports indicate that TRALI can be categorized as having or lacking acute respiratory distress syndrome (ARDS) risk factors. There are two types of TRALI in terms of its pathogenesis: antibody-mediated and non-antibody-mediated. The key initiation steps involve the priming and activation of neutrophils, with neutrophil extracellular traps (NETs) being established as effector molecules formed by activated neutrophils in response to various stimuli. These NETs contribute to the production and release of reactive oxygen species (ROS) and participate in the destruction of pulmonary vascular endothelial cells. The significant role of NETs in TRALI is well recognized, offering a potential pathway for TRALI treatment. Moreover, platelets, macrophages, endothelial cells, and complements have been identified as promoters of NET formation. Concurrently, studies have demonstrated that the storage of platelets and concentrated red blood cells (RBC) can induce TRALI through bioactive lipids. In this article, recent clinical and pre-clinical studies on the pathophysiology and pathogenesis of TRALI are reviewed to further illuminate the mechanism through which NETs induce TRALI. This review aims to propose new therapeutic strategies for TRALI, with the hope of effectively improving its poor prognosis.

## 1 Introduction

**Figure 1:**
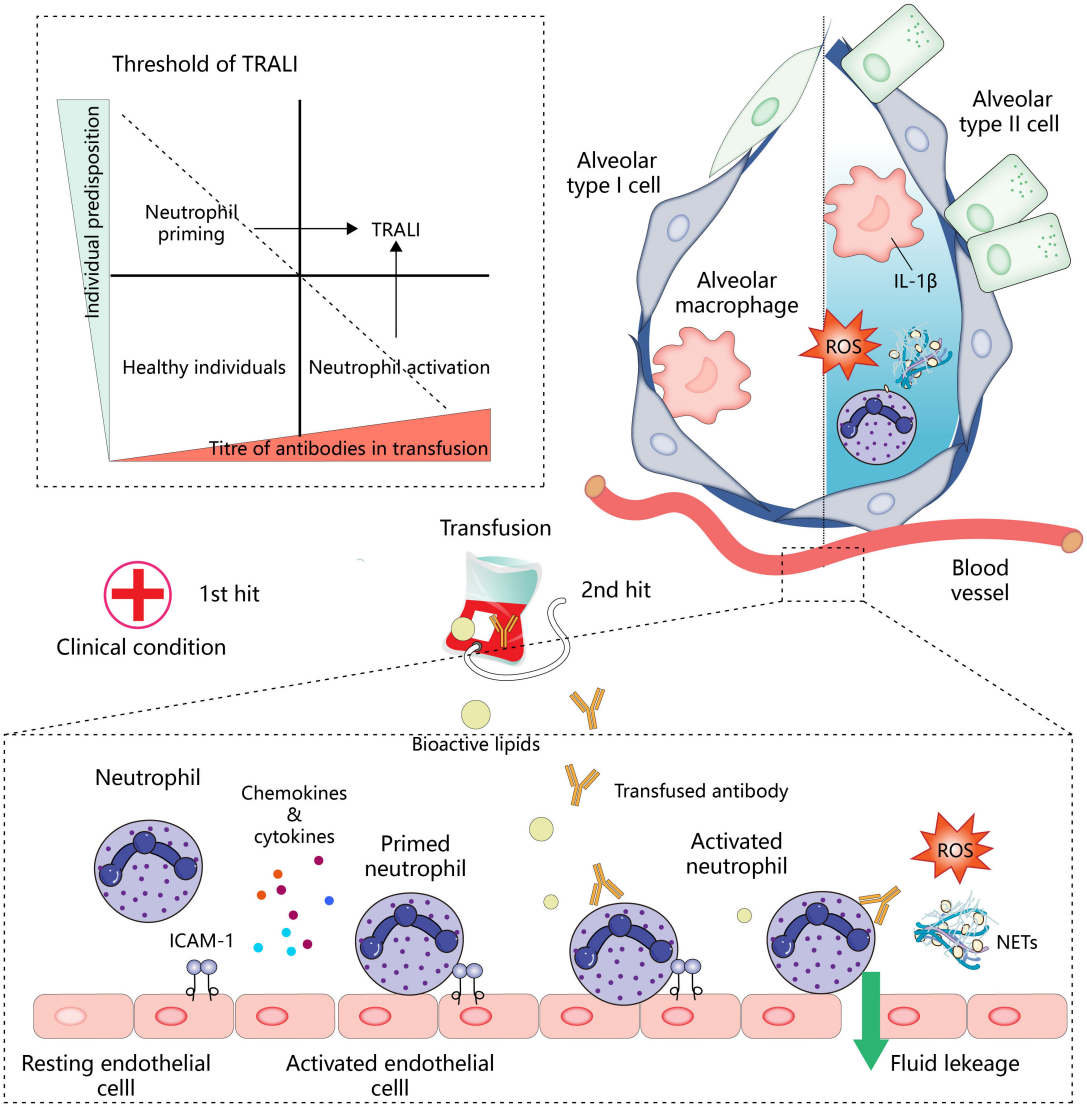
Overview of mechanisms of TRALI development.

Transfusion-related acute lung injury (TRALI) is a severe condition characterized by acute non-cardiogenic pulmonary edema that occurs within 6 hours of transfusion. The latest definition has categorized TRAI into two types: type I (without ARDS risk factor) and type II (with ARDS risk factor or mild existing ARDS) (1). This complication remains a leading cause of transfusion-related fatalities, despite various preventive strategies. Histopathological features of TRALI include endothelial barrier disruption and neutrophil aggregation in the pulmonary vasculature. It is widely assumed that neutrophil respiratory burst releases reactive oxygen species (ROS), resulting in lung endothelial cell injury (2). However, the specific molecular events through which neutrophils are recruited and activated by lung endothelial cells, as well as how neutrophils activate and damage endothelial cells, remain poorly understood. Several models have been proposed to explain the pathophysiology of TRALI. The antibody-mediated hypothesis posits that leukocyte antibodies in transfused blood activate neutrophils, leading to lung endothelial cell damage and subsequent blood penetration into the alveoli, thus triggering TRALI (3). Generally, antibodies causing TRALI include human leukocyte antigen (HLA), human neutrophil antigen (HNA), and corresponding antibodies against the recipient antigen from the donor which, after infusion, initiate neutrophil activation. Controlled clinical studies have identified donor antibody infusion as a recognized cause of TRALI, with blood plasma leukocyte antibodies from female donors posing a particular risk (4). The incidence and mortality of TRALI have been observed to decrease in countries where male donors predominate, though massive transfusion remains an important factor in TRALI whether the blood is from a male or female donor (5). The “two-hit” model suggests that the patient’s underlying clinical condition primes the lung endothelium by increasing the surface expression of adhesion molecules, thereby facilitating the attraction of neutrophils to the lung compartment. Subsequent blood transfusion then acts as the second hit, activating neutrophils via multiple components of the transfused blood products, either directly or indirectly (6). For instance, stored blood often contains higher levels of bioactive lipids, which have been implicated in the two-hit model based on animal models (7, 8). However, conflicting findings have emerged from randomized controlled trials and prospective human studies, indicating that transfusion of stored blood in the presence of endotoxemia does not consistently induce transfusion-related lung injury (9, 10). Furthermore, cases of TRALI have been reported in otherwise healthy individuals, prompting the proposal of a threshold model. This model attributes TRALI development to a combination of patient susceptibility and the number of risk factors present in the blood products. When the mass transfusion of blood products contains a high concentration of antibodies matching the recipient’s antigen, the threshold for neutrophil activation may be surpassed, leading to severe TRALI. This model also highlights specific patient populations as being particularly susceptible to TRALI (11) (**Figure 1**). Despite being described separately, these hypotheses all share the common pathway of neutrophil activation in TRALI. Clarifying the mechanisms underlying neutrophil recruitment and activation, as well as the molecular events governing neutrophil-mediated endothelial cell damage, is critical for advancing our understanding and improving outcomes in TRALI.

Neutrophils are the largest subpopulation of leukocytes in the human body, serving as vital immune defenders through various mechanisms including respiratory burst, phagocytosis, secretion of antimicrobial substances, and the formation of neutrophil extracellular traps (NETs) in response to infectious conditions (12). However, under sterile conditions, extracellular activation of neutrophils may cause damage to normal tissues, such as in the case of TRALI. Although the role of massive ROS in endothelial damage has been described, the ability of neutrophils to form NETs in TRALI is a new piece of knowledge of TRALI pathophysiology. Initially, NETs were viewed as a programmed cell death process, involving the decondensation of nuclear chromatin DNA from activated neutrophils and its fusion with neutrophil granule contents to form a network released into the extracellular space. This network subsequently captures and eliminates microorganisms. However, in the last two decades, our understanding of the formation of NETs has evolved to encompass not only nuclear DNA but also mitochondrial DNA, as well as forms of NETs formation that do not involve cell death. Furthermore, it has been observed that NETs can also form in sterile inflammation, contributing to the progression of pathology (13). As a result, there has been extensive interest in investigating NETs, particularly in the context of sterile inflammation in TRALI. It is crucial to note that the processes governing the formation and function of NETs are subject to both intrinsic and extrinsic immune modulations of neutrophils in order to maintain tissue homeostasis. This delicate balance is essential in mediating between immune defense and tissue damage. Therefore, understanding the interplay between neutrophil extracellular traps and immune regulation may offer valuable insights for potential interventions in the treatment of TRALI resulting from neutrophil activation. Unfortunately, there are very few studies on NETs in TRALI. We therefore tried to summarize the inherent mechanisms of NETs related immune regulation from existing studies, and combined these with the existing evidence and characteristics of TRALI to illustrate the pathobiology and therapeutic implications of NETs in TRALI in order to provide a reference for future studies. In this review, we first provide an overview of the classical pathways and recent insights into NET formation and role of NETs in TRALI, emphasizing the importance of NETs in the development of TRALI. Moreover, we summarize NETs and their key pathways and important mediators of immune regulation, combined with the characteristics of TRALI to discuss the potential role of these immune regulations in the pathophysiological process of TRALI. Finally, we also discuss the translational significance of NET-related immune modulation in TRALI treatment.

Neutrophil activation is central to the development of TRALI. In the two-hit model (below), the first hit, such as the patient’s clinical condition, activates endothelial cells and promotes the expression of intercellular adhesion molecule 1 (ICAM-1) and the secretion of chemokines and cytokines for the recruitment and initiation of neutrophils. Antibodies or other bioactive lipids in the transfusion components represent a second hit, which activates neutrophils, leading to lung endothelial injury and pulmonary edema. In addition, neutrophils infiltrating into the tissues will cooperate with other immune effector cells to amplify inflammation (upper right). Antibody-mediated neutrophil activation resembles the pattern of the second hit. The patient’s predisposition and titer of antibody in transfusion work together to determine whether the threshold for developing TRALI is reached (upper left), and TRALI occurs when these interactions overcome specific thresholds.

## 2 Neutrophil extracellular trap formation

Accumulating evidence now challenges the traditional view of neutrophils as a homogeneous group of responders in the innate immune system. Rather, it suggests that neutrophils consist of more complex subpopulations with distinct functions during infection and inflammation. One subset of activated neutrophils, for instance, is involved in expelling their nuclear contents to clear pathogens, a process known as neutrophil extracellular trap formation. Initially documented by Brinkmann and colleagues, this phenomenon was observed when neutrophils were stimulated with phorbol myristate acetate (PMA), resulting in the release of a network composed of coated histones, neutrophil elastase(NE), myeloperoxidase (MPO), and cathepsin G. Although this process was initially labeled as NETosis, the concept of it being a form of cell death is being challenged by accumulating evidence. Vital NETs, for example, are formed by neutrophils under processes that do not involve cell death.

**Figure 2:**
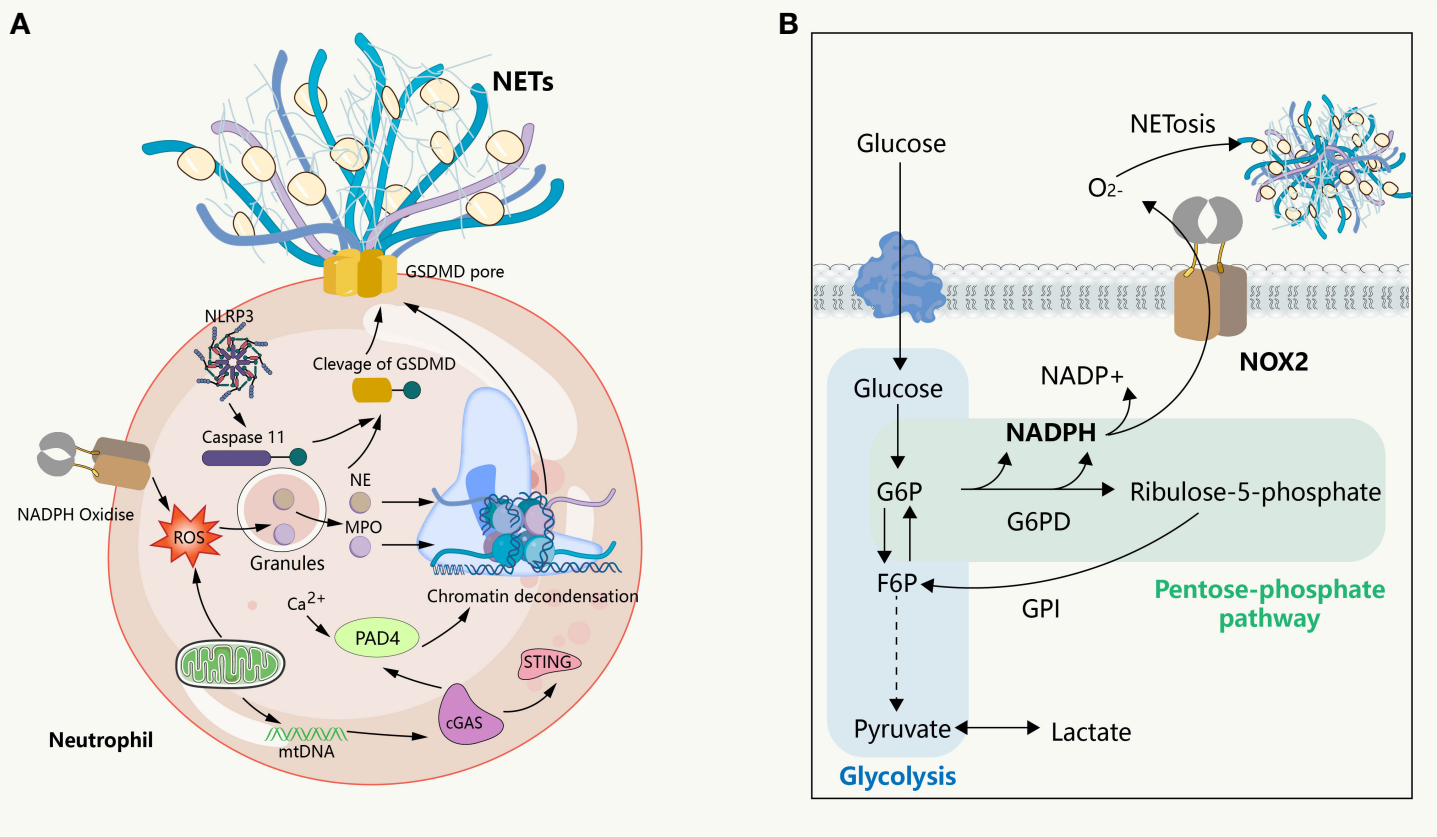
NETs formation and its intrinsic immune regulation of NETs formation. **(A)** NETs formation and the inflammatory pathway intrinsic to neutrophils. NADPH or mitochondrial ROS stimulates the sequential activation of neutrophil MPO and NE, followed by NE entering intranucleus to cleave histones, combined with histone citrullination of PAD 4 promoting chromatin decondensation. Subsequently, under the pore-forming action of GSDMD, the DNA network within the nucleus binds a variety of proteins to release into the extracellular cell. This process is usually affected by the cGAS, NLRP 3 signaling pathway. **(B)** The glucose metabolism in neutrophils. In the resting state, glycolysis is the primary way neutrophils acquire energy, where activated glycolysis is enhanced and a larger fraction of glucose enters the pentose phosphate pathway to support the ROS required to produce respiratory burst and NETs.

NETosis is a clearance mechanism employed by neutrophils in response to excessive or large pathogens and is dependent on the involvement of ROS, MPO, NE, and histone citrullination (**Figure 2**). The formation of NETs occurs through multiple pathways, with optimal ones including the production of ROS by NADPH oxidase, which stimulates MPO to release NE from granules to the cytoplasm, causing degradation of the actin cytoskeleton, impeding neutrophil movement and phagocytosis (14). Subsequently, NE enters the nucleus, where it cleaves histones, unwinds chromatin, and releases DNA. Furthermore, ROS is involved in the activation of protein arginine deaminase 4 (PAD 4), and the spike of cytoplasmic calcium participates in this process. Notably, extracellular ion concentration changes can exert significant effects on NETosis. For instance, elevated sodium ions in the extracellular environment can trigger calcium influx in neutrophils, thereby promoting the formation of NETs in the kidney medulla (15). It is thus imperative to comprehend the regulatory mechanisms associated with ion alterations in specific environments and their impact on neutrophil activation and NET release. The emergence of ion indicators has facilitated the characterization of ion dynamics, offering insights into the regulation of molecular biological processes in distinct physiological settings (16). PAD 4 triggers histone citrullination to promote chromatin decondensation in the neutrophil nuclei (17). The release of NETs into the extracellular space remains inconclusive; however, it has been suggested that cytoplasmic membrane rupture is the dominant view, with gasdermin D (GSDMD), a factor mediating cellular pyroptosis, possibly being involved in the release of the lytic form of NETs. However, subsequent observations question this involvement, as the initial experiment showed this process occurring 3-4 hours after the induction of NETs, whereas subsequent observations suggest that neutrophils can also release NETs within a very short period of time (5-60 minutes) without involving cell death, in a process known as vital NETosis where the plasma membrane is not disrupted and the release of NETs involves nuclear envelope blebbing and cell budding (18, 19). Additionally, in some experiments, NETs consisted of mitochondrial DNA, but not nuclear DNA (20, 21), suggesting that the underlying mechanism could be related to the mitochondria having an independent genome and being an important source of ROS outside of the NADPH oxidase. However, how this form of NETosis releases NETs to the extracellular environment remains unknown. These findings collectively suggest that NETs form through multiple overlapping mechanisms and that the components of DNA networks released into the extracellular space are heterogeneous in different scenarios.

NETs in the extracellular space contain several cytotoxic components, such as DNA, histones, proteases, and highly inflammatory compounds such as myeloperoxidase (MPO), lactotransferrin, LL-37, calprotectin, bactericidal/permeability increasing proteins, and pentraxin 3 (13, 22–26). When released, these components cause indiscriminate damage to both pathogens and bystander cells, thereby potentially contributing to host tissue damage, particularly in the vascular endothelium. Neutrophils release NETs during sterile inflammation, which occurs in response to inflammatory mediators shared with the immune system under sterile inflammatory conditions. This phenomenon is evident in highly vascularized organs such as the lung, where the microvascular endothelium serves as a platform for neutrophil activation and NET release. Furthermore, NETs attacking endothelial cells play a crucial role in the development of pulmonary edema in TRALI. Additionally, DAMPs released from dying neutrophils further activate the innate immune system and amplify the inflammatory response in TRALI. Numerous studies have shown that unregulated NETs play a role in the pathogenesis of various non-infectious diseases, including cancer (27), cardiovascular disease (28), systemic lupus erythematosus (29), rheumatoid arthritis (30), and digestive tract diseases (31), as well as TRALI. However, despite findings indicating the beneficial effects of removing NETs in various diseases, one study reported that NETs promote the resolution of neutrophil inflammation by aggregating and degrading cytokines and chemokines via serine proteases (32). This suggests that NETs may have a dual effect in sterile inflammation. Moreover, in sterile diseases involving neutrophil activation, NETs are not the sole factor causing bystander cell damage, as the activation of ROS and other immune cells by neutrophil respiratory burst also contributes to the process. Consequently, simply targeting NETs may not be the optimal solution for disease treatment. Understanding the interaction between neutrophils and the body, particularly immune regulation, will deepen our understanding of the upstream and downstream effects of NETs and foster the development of improved disease treatments.

## 3 Role of NETs in TRALI

TRALI is characterized by lung endothelial damage and capillary endothelial penetration by activated neutrophils. Neutrophils are the primary component of the lung’s innate immune system and, due to their size compared to lung capillaries, necessitate deformation to overcome the space constraints of the vascular bed. This results in a non-integrin-dependent close contact between neutrophils and vascular endothelium, permitting rapid recruitment and activation of neutrophils under pro-inflammatory conditions, thereby facilitating the rapid onset of TRALI. While it was previously believed that large amounts of ROS released by activated neutrophils were the major factor in endothelial damage, recent studies suggest that NETs also play a significant role in TRALI development. NET structures contain concentrated histones and granule proteins, and high concentrations of locally formed NETs in the pulmonary circulation may enhance the toxicity of neutrophil respiratory burst to endothelial cells (33, 34). The use of DNase1 to degrade NETs has been shown to reduce multiple vascular inflammations, including TRALI, but is not entirely effective in removing all NETs and their cytotoxic substances, such as histones. Circulation of these cytotoxic substances throughout the body post-degradation in the blood may not reach sufficient local concentrations to damage the endothelium. Additionally, extensive interaction between NETs and platelets, in combination with DNA scaffolds, may lead to the formation of thrombi and subsequently cause local ischemic injury in the lung (35, 36). Caudrillier and colleagues (33) first demonstrated NETs’ involvement in endothelial damage in TRALI based on their human specimen-based study, showing that neutrophils in the lung’s microvascular region were undergoing NETosis without forming NETs, despite detecting neutrophils in the alveoli. Another study in humans and mice revealed a substantial accumulation of NETs in the alveoli after anti H-2Kd mAb infusion, but only a small amount in the pulmonary microcirculation, significantly alleviating alveolar aggregation of NETs and decreasing blood oxygen saturation (37). A subsequent study also confirmed that NETs were formed in the alveoli (36). This difference may be attributed to the abundant presence of DNase1 in plasma, which can digest the DNA network of NETs, thereby affording maximum protection to the alveoli from NETs. Furthermore, under TRALI conditions, monocytes/macrophages in the alveoli produce significant amounts of inflammatory mediators, providing strong local stimulation to neutrophils for NET formation.

The induction of NETs can be attributed to various components present in the bloodstream. Studies have demonstrated that the infusion of red blood cells (RBC) can enhance neutrophil adhesion, thereby creating favorable conditions for neutrophil-mediated vascular endothelial injury (38). Furthermore, in long-stored blood, RBC hemolysis can result in hemin accumulation, which has been found to rapidly induce NET formation *in vitro* within 15 minutes, a notably shorter timeframe compared to the 3 hours required for the induction of NETs by PMA (39). Additionally, it has been reported that heme, featuring a porphyrin ring, activates neutrophils to release ROS, thereby potentially triggering subsequent NETosis (40). It can be speculated that the blood components that cause neutrophil activation may also lead to NETosis, but an elusive problem is that there are many factors of neutrophil activation in TRALI, and if NETs are only the product of neutrophil respiratory burst, then the elimination of NETs may not be enough to compensate for the impact of ROS on vascular endothelium and alveolar epithelium damage. Consequently, one may speculate that the blood components responsible for neutrophil activation may also lead to the formation of NETs. However, an unresolved issue pertains to the multitude of factors implicated in neutrophil activation in TRALI, underscoring the importance of resolving this issue for the advancement of therapeutic strategies.

## 4 Interactions of NETosis and immune regulation within neutrophils

### 4.1 Inflammasome signaling pathway

Macrophages and monocytes have widely characterized the lineage and signaling mechanism of the inflammasome. The inflammasome, which acts as a platform for caspase recruitment and activation, triggers the cleavage of the intracellular GSDMD precursor to produce N-terminal fragments (GSDMD-N). These mature N-terminal fragments then bind to the cell membrane in the form of polymers, forming large GSDMD pores that lead to plasma membrane permeabilization and pyroptosis. In contrast, there is a limited understanding of the inflammasome in neutrophils. Although neutrophils express components of the inflammasome and can undergo pyroptosis (41), they are generally resistant to pyroptosis triggered by multiple inducers (42–44). Interestingly, there is a significant interplay between the signaling pathways of inflammasome activation and NETosis, with GSDMD appearing to be the point of interaction between the two pathways. The widely accepted view is that the inflammasome activates GSDMD, leading to the formation of pores in the cell and nuclear membranes that facilitate the release of NETs into the extracellular space (45, 46). Additionally, PAD 4 increases posttranscriptional levels of NLRP3 and ASC, thereby promoting NLRP3 inflammasome/ASC puncta assembly and the production of downstream Caspase-1 and GSDMD-N (46). Furthermore, recent reports have shown that the assembly of ASC puncta occurs at the early stage of NETosis and that the cleavage of the cytoskeleton and nuclear membrane by early formed Caspase-1 likely facilitates the release of NETs (47). However, subsequent studies have indicated that Caspase-11, rather than Caspase-1, is the primary executor of GSDMD cleavage in neutrophils, possibly due to the low expression levels of ASC and Caspase-1 in these cells (48). On the other hand, the PAD 4 enzyme is activated by spiking cytoplasmic calcium and triggers histone citrullination to promote chromatin decondensation, and PAD 4 is activated during caspase-11-driven NETosis, likely due to increased calcium influx through the GSDMD pore (48). However, it has also been proposed that GSDMD cleavage and activation in neutrophils is not independent of caspases but rather dependent on elastase released in granules in neutrophils (49), though neutrophil elastase cleaves several amino acids of GSDMD upstream of the canonical caspase cleavage site, this does not impair the ability of the GSDMD N-terminal inserted membrane to neutralize lysed cells (50). Another study showed that the NE released from the granule cleaves GSDMD, and the cleaved GSDMD forms pores on the granule, promoting further release of NE, which constitutes a feed-forward loop upstream of NETosis (51). Moreover, the pore-forming effect of GSDMD on mitochondria leads to endogenous mtDNA release and further activation of the cGAS-STING signaling pathway, thus promoting the release of NETs and aggravating lung ischemia-reperfusion injury (52). The cGAS also promotes the transcriptional activation of PAD 4 through STING signaling, thereby controlling the formation of NETs (53).

The controversial role of GSDMD in promoting neutrophil NETosis has been the subject of debate, particularly in light of reports indicating its dispensability in PMA-induced NETosis (54). Subsequent studies have reinforced this perspective by demonstrating that GSDMD knockdown in mouse neutrophils still resulted in the formation of NETs with the same kinetics and intensity as those formed by wild-type mouse neutrophils when triggered by classical NETosis inducers such as cytokines and complement factor (55). Therefore, it can be inferred that classical or non-classical stimuli of the inflammasome may not be essential for activating downstream processes of the inflammasome pathway, suggesting a potential disconnect between the requirement for GSDMD and inflammasome-mediated NET formation. Despite previous studies establishing the significance of GSDMD in NETosis, it is important to recognize the involvement of different types of cell-specific signals in NETosis. Consequently, investigating the signaling and regulatory role of GSDMD activation in NETosis will yield critical insights for disease interventions involving neutrophil activation, specifically in conditions such as TRALI. In addition, the release of NETs is also influenced by inflammatory factors from activated neutrophils or other immune cells, a topic we will delve into further. However, within the context of TRALI, the precise immune cell initiators of the immune cascade and the specific role of NETs within this cascade remain open questions.

### 4.2 Immunometabolism of neutrophils

Neutrophils are terminally differentiated leukocytes with the shortest lifespan in the body. They are released from the bone marrow into the blood, function in tissues, and are eventually cleared by other immune cells or returned to the bone marrow as senescent cells. This high degree of transcriptional activity in neutrophils is supported by continuous changes in energy metabolism. Mature neutrophils have minimal mitochondria, with respiratory chain complex inhibitors suggesting little contribution to ATP production (56). Instead, glycolysis is the main pathway by which neutrophils gain energy (57). Glucose enters neutrophils via glucose transporters and is catalyzed into glucose-6-phosphate (G6P) by hexokinase, subsequently entering the glycolytic pathway. When neutrophils were exposed to PMA in a glucose-free medium for 3 hours in previous studies, they lost their characteristic polymorphic nucleus, yet they did not release NETs. However, upon glucose addition, NETs were released within minutes, indicating that the chromatin decondensation phase of NET formation is independent of exogenous glucose, while its release is strictly dependent on glucose metabolism (58). Upregulation of regulatory enzymes in glycolysis, such as 6-phosphofructo-2-kinase/fructose-2,6-bisphosphatase (PFKFB3) and pyruvate kinase M2, is involved in regulating NET formation (59, 60), as neutrophils need to upregulate glycolysis to meet the increased bioenergy requirements for NET formation. In the physiological state, glucose-6-phosphate (G6P) can enter the pentose phosphate pathway (PPP) by the action of glucose-6-phosphate dehydrogenase (G6PD). In this pathway, G6P serves as a substrate for the production of NADPH, which functions as a cofactor and electron donor for NOX. Neutrophils, upon activation, shift their glucose metabolism from the glycolytic pathway to the pentose phosphate pathway, resulting in the subsequent production of NADPH. The NADPH produced acts as a precursor for the generation of superoxide and oxygen radicals under the mediation of NOX. These radicals, in turn, give rise to an abundance of ROS, thereby instigating the respiratory burst of neutrophils and triggering the formation of NETs (57, 61). This assertion finds support in a recent study wherein the inhibition of G6PD curbed the respiratory burst in neutrophils (62). Interestingly, to meet the elevated NADPH demand for neutrophil respiratory burst and the subsequent release of NETs, ribose-5-phosphate, produced through the pentose phosphate pathway, is further metabolized to fructose-6-phosphate (F6P), which is isomerized from G6P by glucose-6-phosphate isomerase (GPI). This process allows for the replenishment of G6P, which can then re-enter the pentose phosphate pathway and yield more NADPH. Flux analysis reveals that during intense oxidative events, neutrophils predominantly utilize the pentose cycle to generate NADPH (63). In contrast, activation of phosphofructokinase 1 to increase glycolytic flux inhibited NET release (64). These findings collectively imply that neutrophils possess a distinctive metabolic adaptability to produce NADPH as needed to facilitate their activation. In contrast, the downregulation of respiratory bursts along with increased NET formation was observed in ARDS induced by COVID-19 (65, 66), suggesting that the pentose phosphate pathway-mediated respiratory burst may not be a conserved mechanism for NETosis. Given the limited glucose availability in lung tissue and local inflammatory regions, the ability of neutrophils to regulate glycogen storage further enhances tissue damage in TRALI, and activation of hypoxia pathways led to increased neutrophil glucose storage and glycolytic capacity, further aggravating the acute lung injury associated with neutrophil activation (67). Moreover, NETs can induce the release of neutrophil granules, producing ROS and subsequent NETosis via NOX2, further aggravating tissue damage (68). Although mitochondria do not appear to be important in neutrophil energy metabolism in neutrophils, their electron transport chain, especially complex III, also leads to mtROS production in a non-NADPH-dependent NETosis. mtROS has been shown to be required for spontaneous NETosis in patients with systemic lupus erythematosus (SLE) (21, 69). The mitochondria-related metabolic changes and mechanisms during neutrophil activation remain an open question, and an understanding of this process will deepen our understanding of NETosis and TRALI. In conclusion, the metabolic status of neutrophils has profound effects on NETosis, and in the context of TRALI, they will further mediate the initiation and progression of tissue damage.

Therefore, in their quiescent and activated states, neutrophils exhibit a high degree of metabolic flexibility that enables them to adapt to phenotypic changes. Although several classical examples of metabolic remodeling associated with respiratory burst and NETosis are recognized, they do not provide a comprehensive metabolic profile of neutrophils. When using inhibitors to target key enzymes in glucose metabolism, it is important to consider the off-target effects of inhibitors and the additional functions of these enzymes beyond classical metabolism. Failing to consider these issues may result in erroneous conclusions. For example, the inhibition of the neutral glycolytic enzyme glyceraldehyde phosphate dehydrogenase (GAPDH) results in the formation of NETs, which is not related to changes in the glycolytic pathway and PPP but is attributed to increased NE activity (**Figure 2**) (70).

## 5 Neutrophil exogenous immune regulator of NETosis

### 5.1 Macrophages

#### 5.1.1 Inflammation boosting

Studies have demonstrated the involvement of lung macrophages in the pathological process of ALI/ARDS resulting from various causes. In inflammatory lesions, macrophage and NETs could provide inflammatory signals such as IL-1β that fuel each other. A study on atherosclerosis has supported this hypothesis, revealing that co-treatment of NETs with cholesterol induces macrophages to produce large amounts of IL-1 β, compared with only a small amount of IL-1 β release using cholesterol alone, implying an important role for NETs in initiating inflammasome activation within macrophages (71). Moreover, in TRALI, the extensive macrophage pyroptosis leads to increased release of IL-1 β, further promoting the release of NETs. Indeed, NETs interact with macrophages through various pathways, supporting positive feedback regulation. NETs can promote the transcription of NLRP 3 through the TLR 4/TLR 9/NFkB signaling pathway (72, 73), and also promote NLRP 3 activation through the ROS/thioredoxin-interacting protein (TXNIP) signaling pathway (72). Additionally, NETs promote the release of other inflammatory factors in macrophages. In renal fibrosis, it has been observed that neutrophil-specific knockdown of GSDMD or Cas-11 reversed the nuclear translocation of p65 in macrophages caused by NETs, and subsequent TGF-1 β release (74). Furthermore, NETs can mediate the activation of NLRP 3 through the TLR 7/TLR 9 signaling pathway (75). The components of NETs contain High-mobility group box 1 (HMGB1), a highly conserved nuclear protein. Studies have shown that NETs initiate the receptor for advanced glycation end products (RAGE) dynamin signaling in macrophages via HMGB1, leading to macrophage pyroptosis (76), suggesting NETs induce macrophage pyroptosis through mutually redundant pathways that amplify local inflammation. NETs also upregulate the activity of the macrophage epidermal growth factor receptor (EGFR), enhance the phosphorylation of Beclin-1 by EGFR, and inhibit autophagosome formation in macrophages, mediating further inflammasome activation (77). As a positive feedback for inflammatory amplification, released IL-1β from macrophages activates the inflammasome in neutrophils, thereby initiating a broader range of NETosis (78). This crosstalk amplifies the local inflammatory response, which, though contributing to a more intense antimicrobial effect in infectious diseases, exacerbates tissue injury in sterile inflammation. Furthermore, NETs cause a significant increase in ROS, promoting the deubiquitination of NLRP3 in alveolar macrophages and, in turn, mediating cell pyroptosis in septic lung injury (79).

The cGAS-STING signaling pathway in macrophages is also involved in recognizing NETs and promoting the NETosis-mediated inflammatory response by releasing type I IFN. Activation of STING by NETs has been observed to promote the pro-inflammatory phenotype transition in macrophages, contributing to stress-induced cardiac remodeling (80) and neuroinflammation (81). Interestingly, when taken up by macrophages via phagocytosis, NETs can escape from the lysosomes and activate the cytoplasmic cGAS, a process that requires the involvement of NE (82).

Based on the evidence described above, manipulating this inflammatory circuit becomes a potential treatment for NET-related diseases, particularly in TRALI, because as a sterile inflammation, sustained activation of neutrophils depends on the involvement of inflammatory factors. It should be noted that the extensive literature on NETs gives the impression that almost all pro-inflammatory molecules induce NETs. However, caution should be exercised in interpreting these results, as the recruitment and activation of neutrophils do not invariably lead to the release of NETs. Moreover, the presence of extracellular DNA in neutrophil activation-associated inflammation cannot be solely attributed to NETs, since pro-inflammatory signaling-induced pyroptosis, necrosis, and apoptosis all result in the release of DNA outside the cell. Additionally, it is worth noting that under certain conditions, blocking inflammatory factors can improve disease progression and reduce NETosis; however, it is important to recognize that the observed positive effects on the disease may be a consequence of the overall reduction in inflammation.

#### 5.1.2 NETs removal

Defects in the regulatory mechanisms responsible for the clearance of NETs may lead to persistent inflammation and deterioration of tissue damage. Physiological concentrations of DNase I in plasma are not sufficient to clear NETs, and, as adjuncts, phagocytes, especially macrophages, are important players in the clearance of NETs. It was previously suggested that NETs internalized by macrophages are degraded in the lysosomal region compartment (83). However, subsequent research revealed that NETs can also be degraded by DNase III outside lysosomes, indicating different nuclease subtypes involved in the degradation process. Notably, while macrophages can degrade NETs, the presence of NETs alone is not adequate to provoke inflammation in macrophages. Conversely, the combined stimulation of NETs may exacerbate LPS-induced inflammation in macrophages, suggesting that damage-associated molecular patterns (DAMPs) or other cytokines may be necessary in the local inflammatory cascade triggered by NETs (84). All types of polarized macrophages possess the capability to clear NETs through the endocytosis of NETs with secreted DNase 1 L3. The clearance of NETs by macrophages may be influenced by increased macrophage pinocytosis following proinflammatory stimulation and altered distribution of DNase by macrophage filopodia production (85). Although a large number of macrophages in the lung tissue are involved in the clearance of NETs during inflammation, proinflammatory macrophages exhibit enhanced NET clearance capacity (85). However, studies based on ARDS patients showed that alveolar macrophages from ARDS patients have a reduced ability to clear NETs, and this process can be reversed by metformin, an activator of AMPK (86), implying that the metabolic reprogramming of proinflammatory cells in a specific tissue context affects the clearance of NETs by macrophages. Additionally, both M1 and M2 polarized macrophages can directly reduce neutrophil NET formation through secreted factors, possibly involving the inhibition of neutrophil ROS (87). Exosomes derived from M2 macrophages can upregulate the endogenous inflammatory “stop signal” lipoprotein A4 (LXA 4) in neutrophils and downregulate the expression of CXCR 2 and ROS in neutrophils, thereby reducing neutrophil migration and NETosis (88). While the specific mechanisms through which macrophages regulate NETosis or NET removal remain unclear, it is evident that the interaction between macrophages and neutrophils is intricate. In some scenarios, they collaborate to establish inflammatory circuits for pathogen clearance or tissue damage, while in other instances, macrophages have an inherent capacity to clear NETs and inhibit neutrophil NETosis. Investigating the interplay between inflammatory progression and resolution involving macrophages and neutrophils is an intriguing area for further study, as it promises to deepen our comprehension of inflammatory diseases, including TRALI.

### 5.2 Dendritic cells and T cells

Plasmacytoid dendritic cells (pDC) express high levels of TLR 7 and TLR 9 that specifically recognize exogenous single-stranded nucleotides in the case of pathogen infection, producing large amounts of type I interferons (89). While pDCs typically do not react to their own DNA, modifications of extracellular DNA can alter this property. For instance, the antimicrobial peptide LL 37 has the capacity to convert inert endogenous DNA into a potent trigger for interferon production in pDCs by binding to DNA and forming aggregated and concentrated structures (90). Furthermore, DNA structures crosslinking multiple proteins within NETs have been demonstrated to carry multiple antimicrobial peptides, forming immunogenic complexes that activate innate pDCs via TLR 9, as evidenced in the autoimmune disease SLE (91). This activation leads to the generation of high levels of interferon-α through TLR 9 ligation, initiating the immune cascade (92–94). Subsequently, these cytokines enable neutrophils to undergo further NETosis, thereby fueling an inflammatory circuit and creating a positive feedback amplification of NETs. Additionally, dendritic cells (DCs) play a role in the clearance of NETs by releasing DNase1-like 3 (DNase1L3) to degrade NETs in the cell supernatants *in vitro* (84). The activation of pDC by NETs seems to bridge innate and adaptive immunity, with NETs initiating the release of inflammatory factors from macrophages, which will further activate helper T cells 17 (TH 17), and subsequently derived IL-17 will drive the chemokines CXCL 1 and CXCL 2 to promote neutrophil recruitment during inflammation (71). In a cigarette smoke-induced emphysema model, it was observed that NETs trigger pDC maturation, subsequently leading to the differentiation of naïve CD4+ T cells into TH17 (95, 96). Although these results imply the involvement of NETs in activating dendritic cells and T cells and promoting innate immunity, the interaction of NETs with adaptive immunity plays an important role in TRALI in the context of acute onset of TRALI.

The involvement of NETs in activating dendritic cells and T cells, as well as promoting innate immunity, has been well-documented in the context of chronic disease. However, it is essential to investigate whether this mechanism is conserved under acute and chronic inflammation, especially in the context of rapid-onset diseases. Therefore, it remains to be elucidated whether the interaction of NETs with adaptive immunity plays a significant role in the acute onset of TRALI.

### 5.3 Complement system

**Figure 3:**
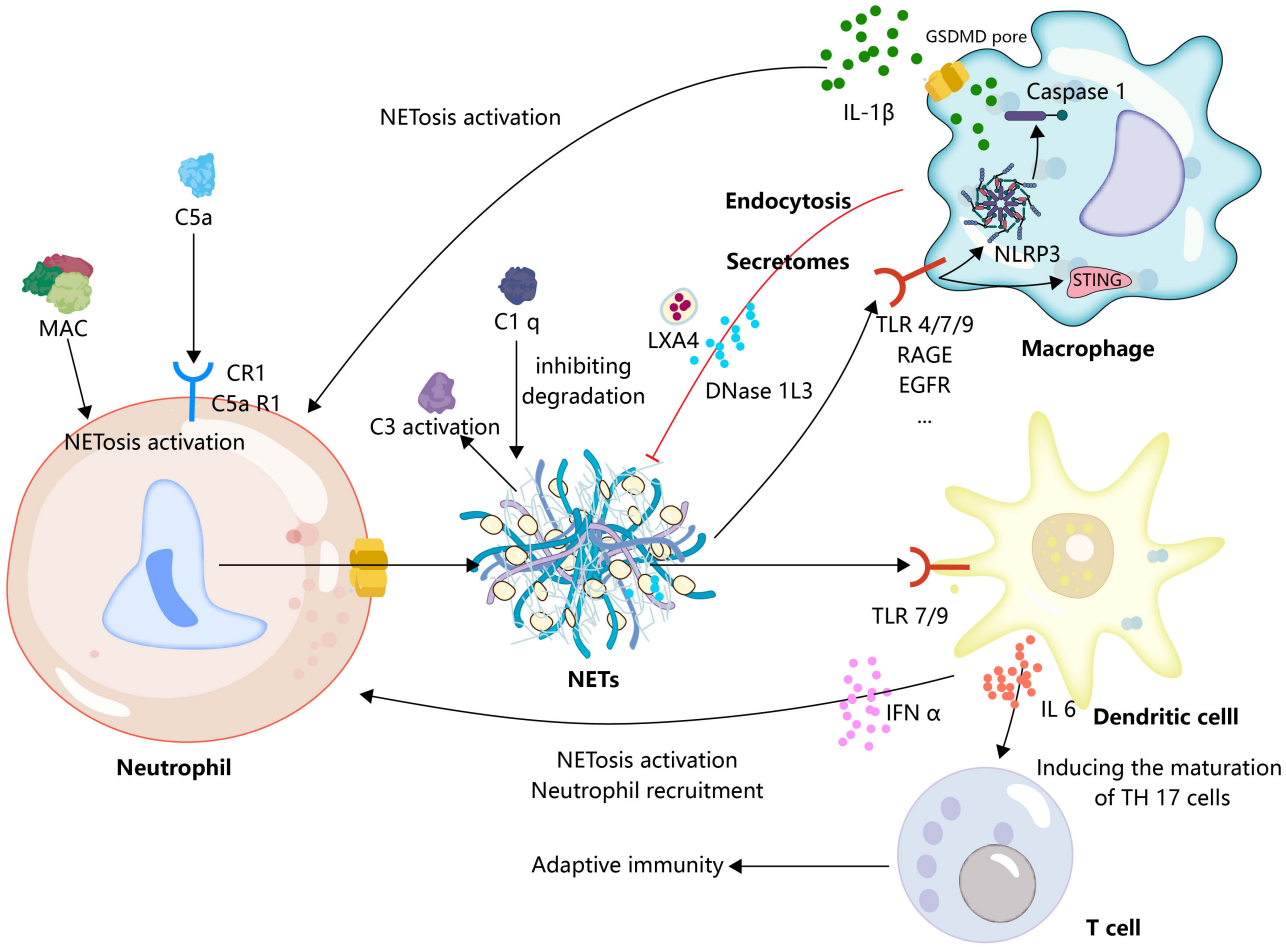
Extrinsic immunomodulation of neutrophils in NETs.

The complement system is a protein reaction system with a precise regulatory mechanism, comprising serine protein cascade enzyme reactions involving continuous cleavage and activation of complement proteins, ultimately leading to target cell lysis through the formation of a membrane attack complex (MAC). In addition, the complement system is involved in the process of opsonization and acts as a danger signal to activate immune cells to initiate immune responses as well as to clear immune complexes (97). The complement system contributes to the induction of NETosis, the ability of S. aureus to induce neutrophil NETosis, and the blockade of complement receptor 1 (CR1) significantly reduces S. aureus-induced release of NETs (98). The process by which C5b-7 is deposited on endothelial cells that physically make contact with neutrophils forms an MAC that transfers to neutrophils and initiates NETosis (99). Pulmonary endothelial activation has been considered to be necessary for the TRALI secondary hit model. In fact, endothelial cell activation may play an important role in complement fixation and activation as recent studies show that antibodies to blood products targeting HLA/major histocompatibility complex (MHC) protein in TRALI are endothelial cells, which subsequently initiate endothelial cells to complement fixation, and depletion of complement reduces antibody-mediated lung injury and NETs (100). In TRALI, a deep exploration of the interactions between the endothelium, complement, and neutrophils will deepen our understanding of this disease. C5aR 1 signaling drives neutrophil NETosis in patients with COVID-19 and drives lung immunopathology in an NET-dependent manner (101). In the mouse TRALI model, C5a acts as a chemoattractant to recruit peripheral neutrophils to the lungs, triggering the formation of NETs in the lung tissue (102). Indeed, as an anaphylaxigin in the complement system, C5a not only recruits neutrophils and initiates upregulation of immune receptors such as TLRs and complement receptors, but also initiates strong NET responses through more profound mechanisms, such as triggering neutrophil NETosis by promoting mitochondrial ROS (103). Furthermore, NETs provide a scaffold for complement activation. Initially, NETs were shown to activate the classical complement pathway by binding complement C1q, and the presence of C1q prevents NETs from being degraded by DNase (104). Subsequent studies showed that NETs also structurally deposited components of the complement alternative activation pathway and that they could activate the complement cascade *in vitro* and in plasma (105, 106). Indeed, neutrophils contain multiple intrinsic components that have been shown to be involved in the activation of the complement system, such as MPO direct binding to acrolein and further induction of C3 activation and interaction of different components of neutrophil granules with properdin (107). When these intrinsic components bind to NETs at NETosis, it can be speculated that they play an important role in the activation of the complement system by NETs. These results imply that the interaction of NETs with the complement system warns the innate immune system to form a pro-inflammatory cycle, and although an attractive model, we still lack enough knowledge to construct this interaction model in TRALI (**Figure 3**).

Neutrophil recruitment and formation of NETs are promoted by activated complement. Concurrently, NETs serve as a platform for complement activation. Additionally, macrophages play a crucial role in providing inflammatory signals, such as IL-1β, to stimulate the formation of NETs. Subsequently, the formation of NETs further triggers macrophages to transition to a proinflammatory phenotype, effectively establishing an inflammatory feedback loop. Furthermore, macrophages are involved in the resolution of NETs through phagocytosis or the secretion of DNAase. In a similar inflammatory context, dendritic cells and neutrophils are capable of reciprocally providing inflammatory signals, such as IFN-α, resulting in the formation of inflammatory circuits. Moreover, dendritic cells activated by NETs can release IL-6, facilitating the maturation of TH17 cells, and thereby linking the innate and adaptive immune responses.

## 6 Therapeutic implications

**Figure 4:**
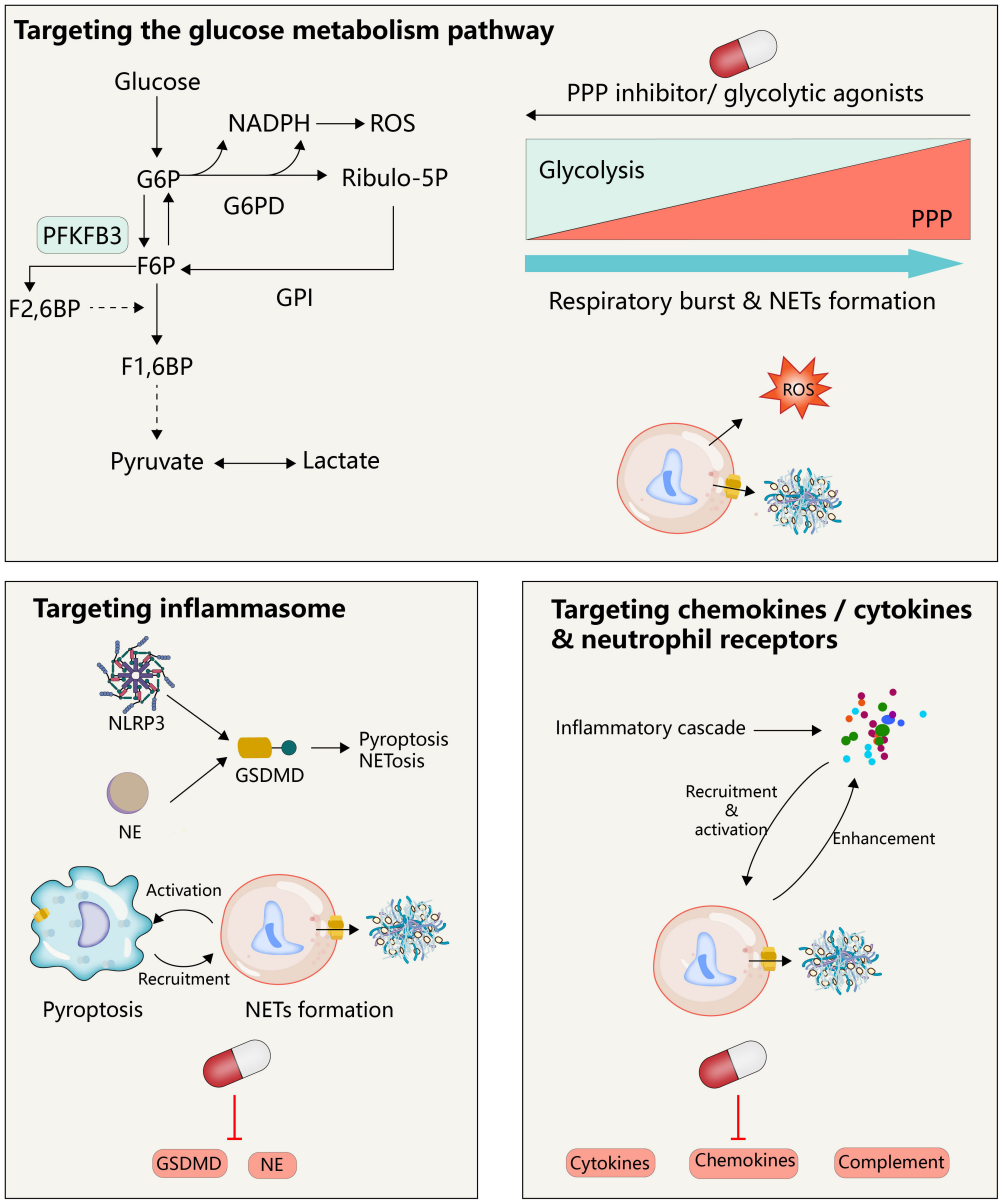
Therapeutic implications.

Given the role of excessive generation or impaired clearance of NETs in tumors, digestive diseases, cardiovascular diseases, the nervous system, and respiratory diseases, several pharmacological pathways have emerged to inhibit NETosis/NETs, including targeted inhibition of NETosis-related PAD 4, NADPH oxidase and ROS generation, and the degradation of DNA in NETs by DNase, which has been well summarized in several excellent other recent reviews (108–110), However, these treatments have some inherent drawbacks, because NETs are only one of the players in inflammation in the complex process of disease progression, and simply targeting NETosis/NETs may not be sufficient to prevent other steps in the immune cascade and tissue damage, such as a strategy using DNase 1 that can only degrade the DNA skeleton of NETs and not alleviate histone-induced damage (111). Here, we will discuss possible therapeutic targets based on immune modulation of NETosis/NETs to attempt to expand the current therapeutic options for TRALI (**Table 1**; **Figure 4**).

**Table 1:** Representative drugs targeting immune regulation in neutrophils.

Principle	Classification	Intervention/Drugs	Mechanisms	References
Targeting the glucose metabolism pathway.	Glycolytic agonist	NA-11	Activates the enzyme PFKL and redirects the metabolic flux from the pentose phosphate pathway back to the glycolytic pathway, consequently inhibiting the NOX 2-dependent oxidative burst in neutrophils.	(64)
	PPP inhibitor	G6PDi	Suppresses G6PD, thus blocking the PPP and respiratory burst	(62)
Targeting inflammasome	GSDMD inhibitor	Disulfiram	Inhibits GSDMD-mediated inflammasome activation	(112, 113)
		ivermectin	inhibits GSDMD oligomerization	(114)
	NE inhibitor	Necrostatin-1	Inhibition of NE activation and the subsequent activation of GSDMD by NE	(115, 116)
Targeting chemokines/cytokines and neutrophil receptors	Human monoclonal antibody against IL-8	HuMab 10F8	Blocks IL-8 to inhibit neutrophil migration	(117)
	Inhibitor of CXCR1 and CXCR2 receptors	Repertaxin	blocks CXCL 1/2 signaling in neutrophils	(118)
	IL-1 receptor inhibitor	IL-1 Ra	Blocks the signaling of IL-1 β in neutrophils	(119, 120)
	Human antibody against N-terminal domain of MK	anti-N-MK	Prevents neutrophil migration	(121)

Targeting the immunomodulation associated with NETs is a potential therapeutic tool for TRALI. Activation of the glycolytic pathway reduces the glucose into the PPP, further inhibiting the NETs associated with neutrophil respiratory burst. Therapeutic strategies targeting GSDMD may inhibit both macrophages and neutrophils, which would not only avoid their independent forms of death in the inflammatory state, respectively, but also avoid their mutual regulation after activation. Neutrophil activity is regulated by multiple cytokines/chemokines, and clearance of these cytokines is a potential therapeutic strategy for targeting NETs.

### 6.1 Targeting glycolytic pathway

Given that neutrophil activation and NET release depend on glycolysis as well as the pentose phosphate pathway, targeting upstream of glycolysis is a promising direction for intervention in TRALI. As mentioned above, the main pathological features of TRALI are neutrophil recruitment activation and endothelial barrier disruption, and inhibition of endothelial activation also prevents barrier system disruption and subsequent infiltration of neutrophil recruitment. It is worth noting that activation of the glycolytic pathway appears to be required for endothelial cell activation (122–124). In lung-related disease, endothelial-specific deletion of the glycolysis regulator PFKFB3 relieves sepsis-related acute lung injury and pulmonary hypertension (125, 126), and underlying mechanisms include inhibition of the glycolytic pathway, inhibiting NF- κB signaling and subsequent reduction of expression of surface adhesion molecules on endothelial cells, and thereby inhibiting neutrophil recruitment (126). Consistently, upregulation of PFKFB increased intracellular lactate levels, followed by activation of NF- κ B, resulting in increased endothelial permeability (127). Furthermore, inhibition of the glycolytic pathway also directly abolished histamine-induced vascular endothelial actin contraction, focal adhesion molecule junction formation, and endothelial barrier disruption (128). From this perspective, targeting glycolysis in TRALI seems to represent a dual benefit, as it not only inhibits endothelial activation-dependent neutrophil recruitment but also suppresses NET formation. It must be noted, however, that the energy biology of endothelial cells at the resting state depends on aerobic glycolysis, and another glycolytic PKM 2 in silent mouse lung endothelial cells depletes ATP required for the normal VE cadherin cycle of endothelial cells (129), leading to basal pulmonary microvascular permeability and pulmonary edema (130). One reason for these observed differences may be the different degrees of glycolysis inhibition. When ATP production is below the lowest amount required to maintain barrier homeostasis, the inhibition of neutrophil activation and NETosis may not be sufficient to counteract the additional lung damage from barrier homeostasis. Thus, the potential of inhibiting glycolysis as a treatment for TRALI needs to be balanced against the risk that it could also trigger TRALI. Furthermore, inhibiting glycolysis downstream may shift metabolic substrates to the pentose phosphate pathway, leading to increased NADPH production, which supports respiratory bursts and NET release. This paradox suggests that while glycolysis is involved in neutrophil activation, targeting the pentose phosphate pathway may be more effective in diseases induced by NETs, including TRALI. Recent studies have explored small molecule inhibitors that target key enzymes in the pentose phosphate pathway, such as G6PDi (62), to inhibit neutrophil respiratory bursts. Moreover, the small molecule NA-11 has been reported to reprogram metabolic flux from the pentose phosphate pathway back to the glycolytic pathway, thus inhibiting NOX2-dependent oxidative bursts in neutrophils (64). Therefore, targeting the pentose phosphate pathway represents a more suitable approach for addressing diseases induced by NETs, including TRALI.

In conclusion, the regulation of glucose metabolism has a profound impact on neutrophil respiratory burst and NETosis. However, the role of glycolysis inhibition in blocking NETosis is still under investigation. Currently, there is no research on TRALI, so a better understanding of the roles of glycolysis and the pentose phosphate pathway in experimental and clinical TRALI models could support the potential role of glucose metabolism as a therapeutic factor for TRALI.

### 6.2 Targeting inflammasome

Alveolar macrophages are key to initiating and resolving lung inflammation, and lung proinflammatory-activated macrophages normally recruit and activate a broader population of immune cells to drive inflammatory diseases, including TRALI (131, 132). Activation of inflammasomes has been shown to be involved in most of the molecular biological processes of proinflammatory macrophage activation and drives multiple pathologic processes of acute lung injury (133–135), thus targeted regulation of the macrophage inflammasome seems a promising direction in the treatment of TRALI. As discussed earlier, inflammasome activation in macrophages promotes the recruitment of neutrophils as well as NET release, meanwhile, activated GSDMD downstream of the neutrophil inflammasome pathway on the nuclear and plasma membranes is critical for NET release, and targeted inhibition of the inflammasome or GSDMD may have unexpected effects in the treatment of TRALI. Sollberger et al. (51) have identified a small molecule LDC7559 that specifically suppresses GSDMD, which hinders membrane localization of GSDMD not only in NETosis in neutrophils but also IL-1 β release in monocytes/macrophages. Disulfiram, a drug approved by the FDA to treat alcohol addiction, has been shown to inhibit GSDMD-mediated inflammasome activation (136). A recent study suggests that pharmacological inhibition of GSDMD by disulfiram reduces the release of NETs from neutrophils and sepsis-mediated multiorgan damage (112). Disulfiram treatment also attenuated the formation of NETs as well as lung immunopathology in a mouse model of severe acute respiratory syndrome coronavirus type 2 infection (113), and also relieved the damage to the endothelium (137). Disulfiram also alleviated the diabetic foot injury mediated by NETs (45). Notably, recent data from animal models suggest that disulfiram can block NET formation and mitigate the pathological phenotype of TRALI (114). This finding highlights the potential for disulfiram as a therapeutic agent in treating TRALI and emphasizes the need for further investigation into the underlying mechanisms of its action in this context. The antiparasitic drug small molecule compound ivermectin was also shown to inhibit GSDMD oligomerization, alleviating the release of NETs and inhibiting *in situ* melanoma metastasis to the lung (138). The favorable effects of other GSDMD inhibitors such as necrosulfonamide (139) and exogenous dimethyl fumarate (115) have been evaluated in macrophages, and the following study seems to re-evaluate the contribution of these compounds in suppressing neutrophil NETs during inflammation. Moreover, targeted regulation upstream of GSDMD can also reduce NET-dependent organ damage. Necrostatin-1 has been shown to reduce inflammation in asthma by inhibiting NET formation (116), and subsequent studies showed that this inhibitory effect is achieved by inhibiting NE activation and subsequent expression of N-GSDMD (117). Thus, the necessity to reevaluate and adjust the use of these drugs is evident in the context of the simultaneous activation of both macrophages and neutrophils in TRALI.

In light of the established safety and efficacy of GSDMD inhibitors in clinical and animal models, it is imperative to reassess and modify the application of these drugs in the context of concurrent activation of macrophages and neutrophils in TRALI. The comprehensive understanding of neutrophil NETosis in TRALI is still incomplete, necessitating clarification on whether NETs in TRALI are released in a GSDMD-dependent manner. This clarification is pivotal before the consideration of GSDMD inhibitors for use.

### 6.3 Targeting chemokines/cytokines and neutrophil receptors

Dysregulation of chemokine/cytokine network has a significant effect on TRALI, characterized by the accumulation of activated neutrophils in pulmonary microvascular regions, and re-regulation of this network contributes to the control of TRALI. In addition to controlling the massive release of the inflammatory cytokines resulting from the activation of inflammatory cells including macrophages and dendritic cells, blocking the ligand-receptor of inflammatory cells is a candidate strategy for targeting inhibiting neutrophil activation and release of NETs. HuMab 10F8 is a full human monoclonal antibody against IL-8, and it effectively neutralizes IL-8-dependent activation of human neutrophils and migration in mid-palmoplantar pustulosis phase (140). Subsequent studies showed that this antibody-based approach of blocking IL-8 to inhibit neutrophil migration similarly inhibited the formation of NETs (141). CXCR 1 and CXCR 2, the receptors blocking IL-8, exerted the same effect, and blocking CXCR 1/2 signaling in neutrophils using the small molecule inhibitor repertaxin protected from organ reperfusion injury (118), and subsequent studies supported that blockade of CXCR 1/2 signaling by repertaxin and pertussis toxin inhibited the release of neutrophil NETs (121). Furthermore, the neutrophil-expressed cytokine midkine mediates neutrophil trafficking and extravasation during acute inflammation, and the antibody anti-N-MK that specifically blocks the N-terminal domain of MK prevents neutrophil migration into the myocardium, thereby alleviating NETosis-driven myocardial inflammation (119). Consistent with this, IL-1 signaling triggered increased expression of leukocyte adhesion molecules on endothelial cells, with reduced neutrophils recruited on the endothelium following administration of the IL-1 α receptor inhibitor IL-1 Ra (120), while blockade of IL-1 β signaling reduced the formation of NETs in breast cancer (142). Blockade of complement signaling represents another strategy to target NETs, and currently, the monoclonal antibody eculizumab targeting the human complement component C5 and the inhibitor avacopan of the C5a receptor have been applied to atypical HUS and ANCA-Associated Vasculitis, respectively (143, 144). Although these drugs have not been evaluated in the clearance of complement activation-associated NETs, based on the extensive interactions between the complement system and neutrophils and NETs, we speculate that these drugs may be potential candidates for TRALI therapy in the future.

It is important to recognize the need for a balanced evaluation of the application of HuMab 10F8, anti-N-MK, eculizumab, and avacopan in the context of TRALI, given the relevance of immunoglobulin-associated TRALI. A recent retrospective study in France highlighted seven cases of TRALI resulting from immunoglobulin infusion, indicating the potential for triggering TRALI through the exogenous infusion of therapeutic antibodies (145). Therefore, before targeting the therapeutic potential of chemokines/cytokines and neutrophil receptors, it is essential to rigorously define the risk of their potential triggering of antibody-mediated TRALI.

## 7 Concluding remarks

It is evident that NETosis does not act independently in inflammatory diseases such as TRALI; rather, it interacts closely with multiple immunomodulatory mechanisms in the human body. Therefore, a comprehensive understanding of these interactions is essential for unraveling the complex processes involved in TRALI initiation, progression, and recovery. Abnormally increased NETs in TRALI exacerbate damage to the endothelium and alveolar epithelium, and also influence other immune cells to produce proinflammatory mediators. This results in the coordination of various immune cells to sustain a detrimental inflammatory cycle. Several dysfunctions associated with TRALI pathophysiological processes create a favorable microenvironment for the formation and release of NETs, such as the feedforward activation of endothelial cells, and the oxygen-rich and glucose-deficient lung environment. Consequently, there is a clear need to explore the multifaceted biological processes of neutrophils and broader immune interactions, with a specific focus on NET formation and further regulation in the context of TRALI.

## Author contributions

YL: Conceptualization, Methodology, Writing – original draft. RW: Conceptualization, Methodology, Writing – original draft. CS: Conceptualization, Methodology, Writing – original draft. SD: Methodology, Writing – original draft. YZ: Methodology, Writing – original draft. KY: Methodology, Writing – original draft. NL: Funding acquisition, Supervision, Writing – review & editing. BW: Funding acquisition, Supervision, Writing – review & editing. QG: Funding acquisition, Supervision, Writing – review & editing.

## Appendix

**Table:** The proteins/genes described in this review.

Proteins/targets	Abbreviations	Gene symbols
Human leukocyte antigen	HLA	*HLA*
Major histocompatibility complex	MHC	
Intercellular adhesion molecule 1	ICAM-1	*ICAM1*
Cathepsin G	/	*CTSD*
Neutrophil elastase	NE	*ELANE*
Myeloperoxidase	MPO	*MPO*
Lactotransferrin	/	*LTF*
LL-37	/	*CAMP*
Calprotectin	/	*S100A8, S100A9*
Pentraxin 3	/	*PTX3*
NOD-like receptor thermal protein domain associated protein 3	NLRP3	*NLRP3*
Apoptosis-associated speck-like protein containing a CARD	ASC	*PYCARD*
Cysteinyl aspartate specific proteinase-1	Caspase- 1	*CASP1*
Cysteinyl aspartate specific proteinase-11	Caspase- 11	*CASP11*
Cyclic guanosine monophosphate-adenosine monophosphate synthase	cGAS	*CGAS*
Stimulator of interferon response cGAMP interactor 1	STING1	*STING1*
6-phosphofructo-2-kinase/fructose-2,6-bisphosphatase	PFKFB3	*PFKFB3*
Pyruvate kinase M2	PKM2	*PKM*
Glucose-6-phosphate dehydrogenase	G6PD	*G6PD*
Glucose-6-phosphate isomerase	GPI	*GPI*
Phosphofructokinase 1	PFK-1	*PFKM/PFKL/PFKP*
glyceraldehyde phosphate dehydrogenase	GAPDH	*GAPDH*
Interleukin- 1β	IL- 1β	*IL1B*
Interleukin- 8	IL-8	*IL8*
Interferon	IFN	*IFN*
Transforming growth factor-β	TGF-β	*TGFB1*
Toll-like receptor 4	TLR 4	*TLR 4*
Toll-like receptor 7	TLR 7	*TLR 7*
Toll-like receptor 9	TLR 9	*TLR 9*
Nuclear factor kappa-B	NF-κB	*NFKB1*
Thioredoxin-interacting protein	TXNIP	*TXNIP*
Beclin-1	/	*BECN1*
High-mobility group box 1	HMGB1	*HMGB1*
Receptor for advanced glycation end products	RAGE	*AGER*
Epidermal growth factor receptor	EGFR	*EGFR*
Adenosine 5’-monophosphate (AMP)-activated protein kinase	AMPK	*PRKAA, PRKAB, PRKAG*
CXC chemokine receptor 1/2	CXCR1/2	*CXCR1/2*
Deoxyribonuclease 1 like 3	DNase1L3	*DNASE1L3*
complement C3b/C4b receptor 1	CR1	*CR1*
VE cadherin	/	*CDH5*
